# Tomato Yellow Leaf Curl Sardinia Virus, a *Begomovirus* Species Evolving by Mutation and Recombination: A Challenge for Virus Control

**DOI:** 10.3390/v11010045

**Published:** 2019-01-09

**Authors:** Juan A. Díaz-Pendón, Sonia Sánchez-Campos, Isabel María Fortes, Enrique Moriones

**Affiliations:** Instituto de Hortofruticultura Subtropical y Mediterránea “La Mayora” (IHSM-UMA-CSIC), Consejo Superior de Investigaciones Científicas, Estación Experimental “La Mayora”. Av. Dr. Wienberg s/n, Algarrobo-Costa, 29750 Málaga, Spain; diazpendon@eelm.csic.es (J.A.D.-P.); soniasc@eelm.csic.es (S.S.-C.); ifortes@eelm.csic.es (I.M.F.)

**Keywords:** *Begomovirus*, genetic diversity, mutation, recombination, tomato, Tomato yellow leaf curl virus

## Abstract

The tomato leaf curl disease (TYLCD) is associated with infections of several species of begomoviruses (genus *Begomovirus*, family *Geminiviridae*) and causes severe damage to tomatoes throughout tropical and sub-tropical regions of the world. Among others, the *Tomato yellow leaf curl Sardinia virus* (TYLCSV) species causes damage in the Mediterranean Basin since early outbreaks occurred. Nevertheless, scarce information is available about the diversity of TYLCSV. Here, we study this aspect based on the sequence information accessible in databases. Isolates of two taxonomically differentiated TYLCSV strains can be found in natural epidemics. Their evolution is mostly associated with mutation combined with selection and random genetic drift and also with inter-species recombination which is frequent in begomoviruses. Moreover, a novel putative inter-strain recombinant is reported. Although no significantly new biological behaviour was observed for this latter recombinant, its occurrence supports that as shown for other related begomoviruses, recombination continues to play a central role in the evolution of TYLCD-associated viruses and the dynamism of their populations. The confrontation of resistant tomatoes with isolates of different TYLCD-associated viruses including the novel recombinant demonstrates the existence of a variable virus x plant genotype interaction. This has already been observed for other TYLCD-associated viruses and is a challenge for the control of their impact on tomato production.

## 1. Introduction

The emergence of viral diseases is a cause of concern because it can result in considerable damage [1,2,3,4]. Among plant viruses, species of the genus *Begomovirus* (family *Geminiviridae*) are economically important phytopathogens that are emerging in warm regions causing severe losses in economically important crops worldwide [5,6,7,8,9,10,11]. Emergence of these viruses has been associated with the emergence of the *Bemisia tabaci* (Hemiptera: *Aleyrodidae*) cryptic species vector complex and its capacity to invade new ecological niches [12,13]. The polyphagous nature of some of these whiteflies favours the movement of viruses latently maintained in native plants to cultivated plants driving the emergence of novel insidious diseases [14].

Begomoviruses as with other species of the *Geminiviridae* family have small twinned (geminate) icosahedral virions that encapsidate circular single-stranded (ss) DNA genomes [15]. Most Old World (OW) begomoviruses are monopartite. The genome of monopartite begomoviruses encodes few open reading frames (ORFs) that are organised bi-directionally in both virus-sense or virus-complementary sense DNA strands and diverge from a noncoding intergenic region (IR) [15]. In the IR, key elements for the replication and transcription of the viral genome are located, including a stem-loop structure that contains the conserved nonanucleotide sequence 5′-TAATATTAC-3′ that forms part of the origin of DNA replication [15,16]. The virus-sense strand encodes the V2 pre-coat protein (V2 ORF), involved in the movement of the virus in plants (Rojas et al., 2001) and the coat protein (CP, V1 ORF), involved in the movement within the plant and also between plants by insect transmission. The virus-complementary sense strand encodes the replication-associated protein (Rep) and the replication enhancer protein (REn) (C1 and C3 ORFs, respectively), that participate in the control of replication, the transcription activator protein (TrAP, C2 ORF), that transactivates expression of virion-sense genes and protein C4 (C4 ORF), that is a pathogenicity determinant [15]. Proteins TrAP, C4 and V2 have also been involved in the suppression of host defence which is mediated by gene-silencing [17,18].

Plant viruses have a high potential to evolve due to their ability for genetic variation through either mutation or genetic exchange by recombination or reassortment of genomic segments [19]. Begomoviruses frequently exploit gene flow provided by recombination as a motor of variation with the resulting modular evolution [20,21] facilitating their ability to challenge environmental changes such as host resistance [22,23,24,25,26,27]. Thus, the extremely high recombination ability of begomoviruses was suggested to be pivotal in their evolution and emergence as a devastating phytopathological problem; recombination is crucial in driving host switches and the further emergence of these viruses [22].

Among monopartite begomoviruses, the *Tomato yellow leaf curl virus* (TYLCV) species is an example of an emergent begomovirus that seriously impacts tomato (*Solanum lycopersicum*) production, it causes the tomato yellow leaf curl disease (TYLCD) epidemics throughout the tropical and sub-tropical regions of the world [28]. A large body of information is available about the genetic diversity, biology and worldwide spread patterns of TYLCV [7,29,30,31,32,33,34,35]. In contrast, much less information is available for other begomoviruses also associated with TYLCD epidemics. This is especially the case for the TYLCV-related monopartite begomovirus species *Tomato yellow leaf curl Sardinia virus* (TYLCSV) reported in the Mediterranean Basin [36,37,38,39]. This virus has been shown to participate in genetic exchanges with TYLCV giving rise to recombinant begomoviruses some of which have a better performance in TYLCD-resistant tomatoes and involved in population displacement in TYLCD epidemics [26,31,40].

Management of begomoviruses is a worldwide challenge; to date, breeding for resistance is considered the best approach to control TYLCD [41,42]. TYLCD-resistant commercial tomato cultivars are available that have been developed from sources of resistance identified and introgressed from several wild tomato species. A number of major resistance loci termed *Ty-1* to *Ty-6*, are available and are used for breeding purposes [41]. Recently, the *Solanum chilense*-derived *Ty-1* and *Ty-3* genes have been demonstrated to be different alleles of the same locus [43], the former being widely used commercially [41].

In this report, we studied the genetic diversity present in TYLCSV populations to get a better insight into the basics of TYLCD-associated virus epidemics and evolution. Data are presented that supports the occurrence of TYLCSV isolates of two taxonomically differentiated strains in natural populations. Also, in addition to mutation combined with selection and random genetic drift, evidence is provided for inter-species recombination as a motor of diversification and evolution of TYLCSV. Moreover, inter-strain recombination was associated with the occurrence of a novel TYLCSV isolate of recombinant nature. The genetic characterisation of this recombinant isolate and construction of an infectious clone allowed to study the biological consequences of such a recombination in terms of host adaptation and resistance-based control.

## 2. Materials and Methods

### 2.1. Plant Materials

The tomato cultivars, tomato breeding lines and tomato relatives used in this study were tomato cv. Moneymaker used as susceptible control, the quasi-isogenic tomato lines homozygous ty-1S (*ty-1*/*ty-1*) (susceptible to TYLCD-associated viruses), heterozygous Ty-1 F1 (*Ty-1*/*ty-1*) and homozygous Ty-1R (*Ty-1*/*Ty-1*) (resistant to both TYLCV and TYLCSV based on gene *Ty-1*) (kindly provided by María José Díez, Universidad Politécnica de Valencia, Spain), the tomato H24 (*Ty-2*/*Ty-2*) and *S. habrochaites* accession EELM-889 resistant to the type strain of TYLCV [44,45], tomato TX 468-RG (kindly provided by Leonardo S. Boiteux, Nacional Centre for Vegetable Crops Research, EMBRAPA-Hortaliças, Brasil) resistant to either bipartite or monopartite begomoviruses controlled by the *tcm-1* recessive gene [46] hypothesised to be an allele of the ty-5 locus [47] and tomato lines susceptible (ty-SkS) and resistant [TY-Sk1 (*Ty-1*/*ty-1*), TY-Sk3 (*Ty-3*/*ty-3*) and TY-Sk13 (*Ty-1*/*Ty-3*)] to TYLCD-associated viruses based on genes *Ty-1* and *Ty-3* (kindly provided by SAKATA Seed Co., Brazil). Also, the common bean cv. Donna (Nunhems, Spain) and a wild *Solanum nigrum* (from IHSM-La Mayora germplasm collection) were used in this study.

### 2.2. Virus Sources

The following infectious clones of isolates of begomovirus species and strains associated with TYLCD in Spain have been described: an isolate of TYLCSV (TYLCSV-[ES-Mur1-92], GenBank accession number Z25751) and isolates of the type (also known as Israel, IL) (TYLCV-IL[ES-Alm-Pep-99], GenBank accession number AJ489258) and Mild (Mld) (TYLCV-Mld[ES-72-97], GenBank accession number AF071228) strains of TYLCV [48,49,50]. Additionally, during routine surveys, field samples from tomato plants exhibiting symptoms of TYLCD (yellowing, upward leaf curling, plant stunting) were collected in 2011 from a commercial tomato crop grown in a greenhouse in Águilas, Murcia (south-eastern Spain), a region where TYLCSV was displaced by TYLCV in tomato [51,52]. Interestingly, the analysis of these samples showed that several of them hybridised only with a probe specific to TYLCSV [49]. Therefore, one sample named ES-Mur-TY2-Tom-11 was considered in this study for further characterisation of the TYLCSV isolate present.

### 2.3. Cloning, Sequencing and Construction of an Infectious Clone of Isolate [ES-Mur-TY2-Tom-11]

The total DNA extracted from young leaf tissues of the sample ES-Mur-TY2-Tom-11 using a modified CTAB method [53] was used to amplify the circular DNAs present by means of a rolling-circle amplification (RCA) reaction using φ29 DNA polymerase (TempliPhi kit, GE Healthcare, Chicago, USA), according to the manufacturer’s instructions. RCA-amplified products were partially digested with the single cutting *Bam*HI restriction endonuclease and tandem repeat fragments were purified from agarose gel (QIAquick Gel Extraction Kit, QIAGEN, Hilden, Germany) and ligated into the *Bam*HI cloning site of pCAMBIA 0380 vector (Cambia, Canberra, Australia). A virus-specific clone (pMur-TY2-Tom-11) containing an insert of about 5.6 kb (a putative dimeric 2-mer clone with a tandem repeat of two full-length copies) was selected to derive the full-length sequence of the cloned isolate named [Spain-Murcia-TY2-Tomato-2011] (acronym ES-Mur-TY2-Tom-11). A primer-walking strategy was employed to obtain the full-length viral genomic sequence (GenBank Accession No. KC953604). An infectious clone of the isolate [ES-Mur-TY2-Tom-11] was obtained by transferring the pCAMBIA clone pMur-TY2-Tom2011 into the *Agrobacterium tumefaciens* strain LBA4404.

### 2.4. Virus Inoculation, Detection and Symptoms Evaluation

For *A. tumefaciens*-mediated inoculation (agroinoculation), liquid cultures of *A. tumefaciens* were pelleted and resuspended in an agroinoculation buffer [10 mM MgCl_2_, 10 mM 2-(*N*-Morpholino) ethanesulfonic acid (MES) pH 5.8 and 2.25 mM acetosyringone] to a final OD of 1.0 at 600 nm before stem puncture inoculation [54]. Plants inoculated with *A. tumefaciens* carrying the empty vector were used as mock-inoculated controls. For *B. tabaci*-mediated inoculation, viruliferous whiteflies were obtained by providing adult individuals of the *B. tabaci* Mediterranean species with a 48-h acquisition access period (AAP) on systemically infected young leaves of tomato cv. Moneymaker plants agroinoculated three weeks earlier. After the AAP, groups of viruliferous whiteflies were transferred to healthy tomato test plants (25 whiteflies per plant) for a 48 h inoculation access period (IAP) using clip-on cages. Then, after insecticide treatment, inoculated plants were maintained in an insect-proof growth chamber (26 °C day and 18 °C night, 70% relative humidity, with a 16-h photoperiod at 250 mol s^−1^ m^−2^ photosynthetically active radiation) until the analysis of the presence of the virus and visual observation of the development of TYLCD symptoms. The presence of viral DNA was analysed in each test plant by hybridisation of tissue blots from young leaf petiole cross sections using probes specific to TYLCV and TYLCSV as described by Monci et al. [24]. Also, when needed, the presence of the virus was analysed by PCR using the primer pair MA272 (5′-CTGAATGTTYGGATGGAAATGTGC-3′, corresponding to nt 2342–2365 on GenBank X61153) and MA273 (5′-GGTTCGTAGGTTTCTTCAACTAG-3′, complementary to nt 225–247 on X61153) [52]. Symptom severity was evaluated using a 0–5 arbitrary scale, where 0 was assigned to asymptomatic plants and 5 was assigned to severe TYLCD symptoms. Curves of the progress of the symptom were constructed and the area under the symptom progress curve (AUSPC) was determined using the following formula: AUSPC = Σi([xi + x1 + i]/2)ti, where xi = mean value of disease score at date i and ti = time (in days) between scoring date i and scoring date i + 1 [55]. TYLCD-like disease severity for each line was estimated from virus infected plants according to the following formula: disease severity index (DSI): (Σ(0a + 1b + 2c + 3d + 4e + 5f)/(a + b + c + d + e + f) × 5) × 100 where a, b, c, d, e and f are the number of plants that displayed each disease severity score.

### 2.5. Analysis of Sequence Data

The analysis of the genetic relationships among isolates of TYLCSV was performed based on the full-length sequences available as of the 19th of September 2018 in the National Centre for Biotechnology Information (NCBI)-GenBank database and/or listed by the *Geminiviridae* Study Group of the International Committee on Taxonomy of Viruses (summarised in Table 1). Sequences were aligned using the Multiple Sequence Comparison by Log-Expectation (MUSCLE) computer software [56]. Pairwise nucleotide identity comparisons were calculated using the Sequence Demarcation Tool program (SDT; Version 1.2) [57] with the MUSCLE [56] option for sequence alignment. Phylogenetic relationships were represented by means of a tree built using the neighbour-joining method based on the Tamura–Nei model available in the Molecular Evolutionary Genetics Analysis (MEGA) 5.0 software [58]. One thousand bootstrap replicates were used to assess the robustness of the final tree topology.

The detection of potential recombinant sequences, identification of putative parental sequences and localisation of possible recombination breakpoints was based on the plot similarity analysis performed with the SimPlot 3.5.1 software with the Kimura 2-parameter distance model [59] and the recombination detection program RDP4 with standard Bonferroni correction and default settings [60]. When needed, portions of sequences that diverged from that of the isolate used as the query in plot similarity comparisons were used for sequence similarity search using the Basic Local Alignment Search Tool (BLAST) [58] to locate nucleotide sequences of related begomovirus isolates.

## 3. Results

### 3.1. TYLCSV Population Is Composed of Isolates of Two Differentiated Strains

**Figure 1:**
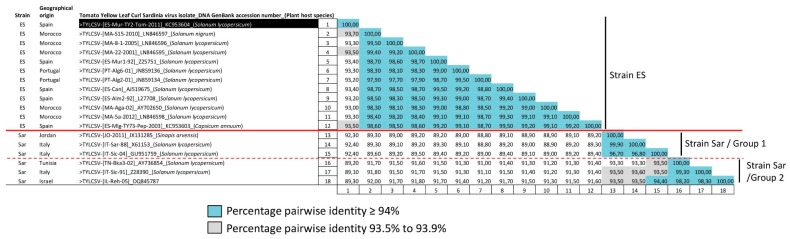
Two-dimensional identity matrix between full-length tomato yellow leaf curl Sardinia DNA genome sequences. The pairwise nucleotide sequence identity percentages is shown between full-length DNA sequences of tomato yellow leaf curl Sardinia virus (TYLCSV) isolates available in database as of the 19th of September 2018, calculated using the Sequence Demarcation Tool program (SDT; Version 1.2) [57] with the MUSCLE [56] option for sequence alignment. Virus isolate (acronyms according to data compiled in Table 1), GenBank accession number and plant host species from which the isolate was obtained are indicated; also, the geographical origin of the isolate is compiled. Following the International Committee on Taxonomy of Viruses (ICTV) current guidelines for begomoviruses the threshold used for strain demarcation is 94% (with percent identities rounded to the nearest full percentile as suggested) [68]; percentages of nucleotide identity > 94%, are shown in blue colour and between 93.5% and 93.9% are shown in grey colour. Based on ICTV criteria, two strains (named Spain, ES and Sardinia, Sar) can be differentiated; isolates that correspond to different strains are shown in the first column (solid red line separation). The dashed red line separates two groups of isolates within the strain Sar (named Group 1 and Group 2) slightly different based on sequence identity. The TYLCSV isolate [ES-Mur-TY2-Tom-11] reported in the current study is highlighted with a black box.

**Figure 2:**
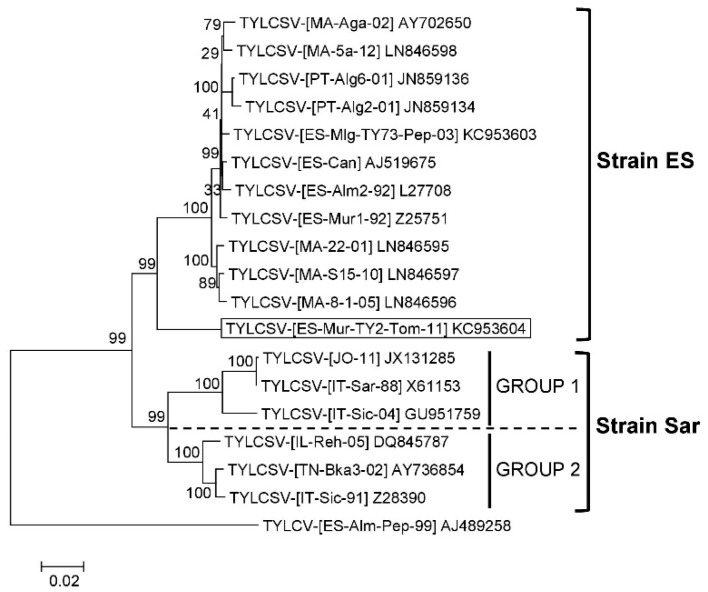
Phylogenetic relationships among full-length DNA genome sequences of tomato yellow leaf curl Sardinia virus (TYLCSV) isolates. Isolates available in databases as of the 19th of September 2018 were considered (acronyms and GenBank accession numbers according to data compiled in Table 1). The phylogenetic tree was built using the neighbour-joining method and bootstrap (1000 replicates) values are expressed as percentages; isolates of TYLCSV that correspond to strains Spain (ES) and Sardinia (Sar) are indicated. The scale bar indicates 0.02 nucleotide substitutions per site. The sequence of the isolate [ES-Alm-Pep-99] of tomato yellow leaf curl virus (TYLCV) (GenBank Accession Number AJ489258) was included as outgroup and the TYLCSV isolate [ES-Mur-TY2-Tom-11] reported in the current study is boxed.

A total of 18 full length genome sequences were available in the databases (including the one reported in this work) as of the 19th of September 2018 for TYLCSV isolates, all from different Mediterranean Basin countries (Table 1). Pairwise nucleotide sequence comparisons of these sequences revealed that following taxonomic criteria for strain demarcation currently established for begomoviruses (94% sequence threshold) by the *Geminiviridae* Study Group of the International Committee on Taxonomy of Viruses (ICTV) [68] TYLCSV isolates grouped into two differentiated strains (Figure 1). No such strain differentiation in TYLCSV species is currently done, however, by the ICTV [68] (current ICTV Report on Taxonomy of Viruses https://talk.ictvonline.org/ictv-reports/ictv_online_report/ and latest *Begomovirus* isolate list of the *Geminiviridae* Study Group of the ICTV). According to names proposed in previous virus taxonomy publications of the ICTV [69], these two strains were named Spain (ES) and Sardinia (Sar). The strain Sar contained isolates of the previously proposed strains “Sar” and “Sic” that based on the present study merged in a single strain. The name (Sar) of the strain associated in previous studies to the first reported TYLCSV isolate [62] was conserved in this case. The strain ES consists of isolates reported from Spain, Portugal and Morocco, whereas the strain Sar comprises isolates from Italy, Israel, Jordan and Tunisia. High nucleotides identities were observed among the genomes of isolates grouped in the strain ES (≥98%). In contrast, two differentiated groups (named Group 1 and Group 2, see dashed red line separation in Figure 1) could be differentiated based on the comparison of the genomes of TYLCSV strain Sar isolates. High nucleotide sequence identities (≥97%) were observed between isolates within each group of the strain Sar whereas nucleotide sequence identities close to the threshold of strain differentiation (<94% identity) were observed between isolates of these two groups. Phylogenetic relationships among the TYLCSV isolates (Figure 2) support the relationships observed in the SDT comparison, with two clearly differentiated clades grouping separately isolates of strain ES or strain Sar and two additional clades within strain Sar separating isolates of Group 1 or Group 2. Therefore, isolates of two taxonomically differentiated strains are present in the TYLCSV population in natural epidemics.

### 3.2. TYLCSV Is Evolving by Mutation and Recombination

**Figure 3:**
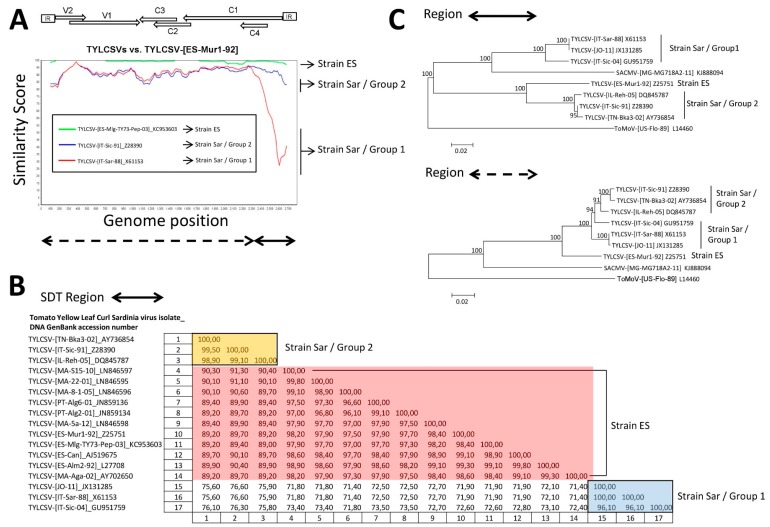
Genome comparison of the isolates of tomato yellow leaf curl Sardinia virus (TYLCSV). Isolates available in the databases as of the 19th of September 2018 were considered (acronyms and GenBank accession numbers according to data compiled in Table 1). (**A**) A plotsimilarity diagram (scanning window 200 nt) comparing the full-length DNA sequences of representative isolates of TYLCSV from stains Spain (ES) and Sardinia (Sar) (Group 1 and Group 2) using the nucleotide sequence of isolate TYLCSV-[Spain-Mur1-92] (GenBank Accession Number Z25751) of. strain Spain (ES) as the query. Double arrow lines at the bottom indicate the most similar (dashed line) and dissimilar (solid line) genomic regions among all isolates. Positions of the open reading frames and of the intergenic region (IR) are indicated at the top of the figure. (**B**) Two-dimensional identity matrix between the nucleotide sequences of the genomic region of TYLCSV isolates delimited by the solid double arrow line of panel (A) calculated using the Sequence Demarcation Tool program (SDT; Version 1.2) [57] with the MUSCLE [56] option for sequence alignment. (**C**) Phylogenetic relationships among DNA sequences comprised in the genomic regions delimited by the double arrow lines at the bottom of panel A of TYLCSV isolates of the strain Sar, one representative isolates of strain ES (isolate TYLCSV-[ES-Mur1-92]) and the corresponding sequence of DNA-A component of isolate [MG-MG718A2-11] of South African cassava mosaic virus (SACMV). The tree was constructed using the neighbour-joining method. Bootstrap (1000 replicates) values are expressed as percentages. The scale bar indicates 0.02 nucleotide substitutions per site. The equivalent sequence of the DNA-A of isolate [US-Flo-89] of the begomovirus tomato mottle virus (ToMoV) was included as outgroup. Positions of isolates of strains Spain (ES) and Sardinia (Sar) (Group 1 and Group 2) are indicated.

Plotsimilarity comparison of the full-length genome sequences of TYLCSV isolates available in databases (Figure 3A), showed a close relationship among the sequences of strain ES isolates. In contrast the sequences of isolates of strain Sar were more dissimilar to those of isolates of strain ES [shown in Figure 3A for representative TYLCSV isolates from stains ES and Sar (Group 1 and Group 2)]. In this latter case, strain Sar/Group 2 isolates exhibited point mutation divergence from strain ES isolates throughout their genome. Interestingly, strain Sar/Group 1 isolates exhibited a close relationship with strain Sar/Group 2 isolates except for a greatly dissimilar region corresponding to the IR and the 5′ third of the C1 ORF (shown by a solid double arrow line at the bottom of Figure 3A). A closer analysis revealed that nucleotide identities among sequences of TYLCSV isolates in this latter region resulted in three differentiated groups of isolates (Figure 3B), one that corresponds to the strain ES isolates and the other two groups that differed among them and from strain ES isolates that comprised strain Sar/Group 1 and strain Sar/Group 2 isolates. Interestingly, when a BLAST search was done, a strong relatedness was detected for this genomic region of strain Sar/Group 1 isolates with the equivalent sequence of an isolate of the begomovirus species *South African cassava mosaic virus* (SACMV) (isolate [MG-MG718A2-11], GenBank accession number KJ888094). Therefore, this suggests that this genomic region of strain Sar/Group 1 isolates might have been acquired from a SACMV-related ancestor by genetic exchange through recombination, as already suggested [27]. Statistical support was obtained by using the recombination detection program RDP4 (Supplementary Materials Table S1). Evidences were also obtained from the incongruences in the phylogenetic position of isolates of strain Sar/Group 1 when TYLCSV isolate relationships were analysed separately for the two regions of the genome (Figure 3C). The association of isolates of strain Sar/Group 1 with SACMV occurred when sequences in the small differing genome portion (solid double arrow region shown at the bottom Figure 3A) were compared, whereas they grouped together with strain Sar/Group 2 isolates when comparing sequences in the other portion (dashed double arrow in Figure 3A) of the genome. This supported different phylogenetic origins of both sequence regions in isolates of strain Sar/Group 1 compatible with inter-species recombination.

### 3.3. A TYLCSV Recombinant Isolate Detected in Spain Putatively Resulted from a Genetic Exchange between Isolates of Strains ES and Sar of TYLCSV

**Figure 4:**
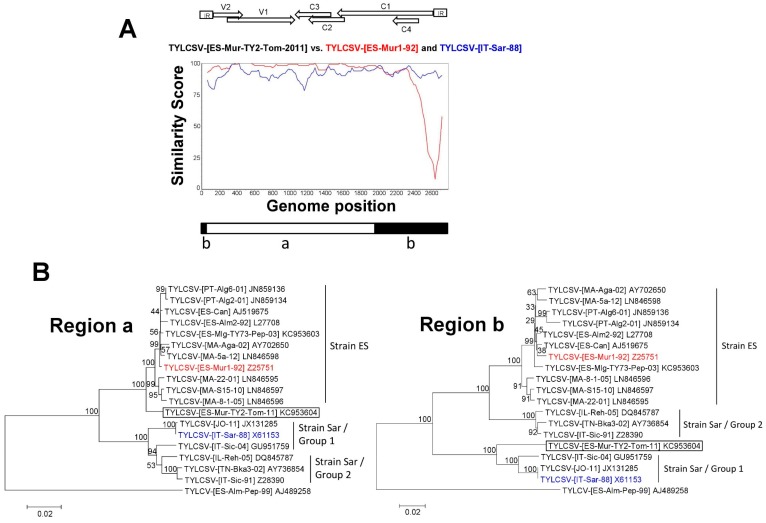
(**A**). Plotsimilarity diagram (scanning window 120 nt) comparison of the full-length DNA genome sequence of the recombinant tomato yellow leaf curl Sardinia virus (TYLCSV) isolate [ES-Mur-TY2-Tom-11] (GenBank Accession No. KC953604) characterised in the current study with those of representative isolates of parental viruses putatively involved in the recombination: isolates TYLCSV-[ES-Mur1-92] (GenBank Accession No. Z25751) and TYLCSV-[IT-Sar-88] (GenBank accession No. X61153) of strains Spain (ES) and strain Sardinia/Group 1 (Sar/Group 1), respectively. The regions putatively involved in the recombination event deduced based on the results from the recombination detection analysis are indicated by open and filled boxes (regions “a” and “b,” respectively) at the bottom of the figure. Positions of the open reading frames and of the intergenic region (IR) are indicated at the top of the figure. (**B**). Phylogenetic relationships among DNA sequences of TYLCSV isolates in regions “a” and “b” of the genome defined in panel (A). The trees were constructed using the neighbour-joining method. Bootstrap (1000 replicates) values are expressed as percentages. The scale bar indicates 0.02 nucleotide substitutions per site. Virus species acronyms are used according to Table 1 and GenBank accession number is indicated for each isolate included in the analysis; positions of isolates of strains Spain (ES) and Sardinia (Sar) (Group 1 and Group 2) are shown. The equivalent sequences of the genome of isolate [ES-Alm-Pep-99] of tomato yellow leaf curl virus (TYLCV) (GenBank AJ489258) were included as outgroups. Representative isolates of the parental TYLCSV strain ES and strain Sar/Group 1 viruses putatively involved in the genetic exchange resulting into the recombinant TYLCSV isolate [ES-Mur-TY2-Tom-11] reported in the current study (boxed) are shown in red and blue types, respectively.

Tomato sample ES-Mur-TY2-Tom-11 was obtained from a TYLCD-symptomatic plant collected during routine surveys conducted in 2011 in Águilas (Murcia, south-eastern Spain). Preliminary hybridisation analyses showed that this sample hybridised only with a TYLCSV-specific probe. This was surprising because in this geographical area almost a complete displacement of TYLCSV by TYLCV has occurred [52]. Therefore, one isolate [ES-Mur-TY2-Tom-11] derived from this sample was further characterised here. The complete genome sequence of this isolate was determined from the full-length genome fragment cloned in pMur-TY2-Tom2011 clone. The sequence comprised 2775 nucleotides and has been deposited in the EMBL/GenBank Nucleotide Sequence Database under Accession No. KC953604. The genome organisation of the isolate [ES-Mur-TY2-Tom-11] was similar to that reported for other Old World monopartite begomoviruses, with two ORFs in the virion-sense strand and four ORFs in the complementary-sense strand separated by the IR. The IR contains the nonanucleotide 5′-TAATATTAC-3′ sequence conserved in begomoviruses within a putative stem-loop structure. Comparison of the obtained sequence with sequences available in the databases showed identities above 91% with that of other isolates of TYLCSV (Figure 1). Therefore, following ICTV species demarcation guidelines for isolates in the genus *Begomovirus* of the family *Geminiviridae* (91% identity threshold used for species demarcation) [68], the isolate [ES-Mur-TY2-Tom-11] belongs to the TYLCSV species of TYLCD-associated begomoviruses. Also, based on sequence identities, this isolate was classified within the strain ES of TYLCSV (full-length sequence identity >93.5%) reported here (Figure 1). However, a plotsimilarity comparison of the full length genome sequence of this isolate with that of a representative isolate (isolate TYLCSV-[ES-Mur1-92] of strain ES, Figure 1) of the TYLCSV described in Spain until this moment (red line in Figure 4A) showed that a close similarity existed between them except for a region corresponding to the 5′ part of the Rep (C1 ORF) (comprising C4 ORF) and the IR (region “b” Figure 4A), suggesting a recombinant origin. In contrast, a closer relationship was found for this latter region with that of a representative isolate (isolate TYLCSV-[IT-Sar-88]) of the strain Sar/Group 1 of TYLCSV (blue line in Figure 4A), a strain not reported in Spain. Six methods implemented in the RDP4 recombination analysis program supported the evidence of a recombinant origin for isolate [ES-Mur-TY2-Tom-11] when reported TYLCSV isolates (Table 1) where included in the analysis (Table 2). Isolate TYLCSV-[ES-Mur1-92] which was previously reported in Spain and belongs to the strain ES of TYLCSV was identified as the closest major parent and the closest minor parent for the fragment spanning the IR and the 5′ proximal part of the Rep gene (nt coordinates 1951 to 56 in GenBank KC953604) was isolate TYLCSV-[IT-Sar-88] (GenBank X61153), which belongs to the strain Sar/Group 1 of TYLCSV (Figure 1). The RDP analysis supported that isolate [ES-Mur-TY2-Tom-11] is a recombinant isolate putatively resulted from a genetic exchange that merged sequences from isolates of strains ES and Sar/Group 1 of TYLCSV. Moreover, separated phylogenetic comparisons of sequences in regions “a” and “b” involved in the recombination event (Figure 4) of TYLCSV isolates showed topological position incongruence for isolate [ES-Mur-TY2-Tom-11], that grouped together with strain ES (region “a”) or strain Sar/Group 1 (region “b”) isolates depending on the region compared (Figure 4B). The latter again supports different evolutionary origins for both sequence regions of isolate [ES-Mur-TY2-Tom-11]. Therefore, based on the data presented, the genome of this isolate has a modular organisation resulted from its putative inter-strain recombinant origin.

Interestingly, in Figure 4B, an additional change in the phylogenetic topological position was also observed for isolates of strain Sar/Group 2 that grouped with isolates of strain Sar/Group 1 (region a) or strain ES (region b) depending on the genomic region compared. As observed in the plotsimilarity analysis shown in Figure 3A, the sequences of these isolates resemble those of strain Sar/Group 1 isolates for most of their genomes (dashed double arrow at the bottom) except for the region (solid double arrow at the bottom) spanning the IR and the 5′-end of the Rep gene in which they are more closely related to those of isolates of strain ES. These differences determined the incongruence in the topological position of these isolates depending on the region compared. Therefore, as indicated above, strain Sar/Group 2 isolates belong to the TYCSV strain Sar ancestor involved in the genetic exchange that resulted into the strain Sar/Group 1 recombinant isolates.

### 3.4. An Infectious Clone of TYLCSV Recombinant Isolate [ES-Mur-TY2-Tom-11] Efficiently Infects Tomato and Solanum Nigrum Plants

A dimeric infectious clone of isolate [ES-Mur-TY2-Tom-11] was obtained by transferring the clone pMur-TY2-Tom2011 into *A. tumefaciens*. Infectivity of the infectious clone was confirmed by agroinoculation of tomato cv. Moneymaker plants, with a 100% infection success (10 plants systemically infected of 10 plants inoculated in the two assays performed) (Table 3) and detection of the presence of virus in young non-inoculated tissues by hybridisation from 14-day-post inoculation (dpi). Also, inoculated plants exhibited typical TYLCD symptoms of infection (from 28 dpi) similar to those observed in the field plant from which the isolate was derived. Moreover, when using agroinoculated plants as the virus source for *B. tabaci* transmission, the virus was efficiently acquired and transmitted to healthy tomato plants (8 plants systemically infected out of 8 inoculated using 25 viruliferous whiteflies per test plant). *B. tabaci*-inoculated plants also exhibited typical TYLCD symptoms of infection from 28 dpi. The identity of the inoculated virus in newly-emerged non-inoculated apical leaves of infected test plants was confirmed by PCR amplification (primer pair MA272/MA273) and direct sequencing of the fragment amplified from one agroinoculated and one *B. tabaci*-inoculated plant. No infection, symptom development, or virus transmission was observed for tomato plants agroinoculated with an empty vector. Therefore, these results indicate that a fully biological active infectious clone of [ES-Mur-TY2-Tom-11] was obtained.

The ability of TYLCSV[ES-Mur-TY2-Tom-11] to infect common bean and *S. nigrum* plants was studied because differences have been reported in these host plants for TYLCD-associated viruses and derived recombinants [24,70]. As shown in Table 3, the recombinant TYLCSV[ES-Mur-TY2-Tom-11] similarly to the control TYLCSV isolate of the strain ES used, efficiently infected plants of *S. nigrum*. In contrast, the TYLCSV isolate of the strain ES previously described in Spain was unable to systemically infect common bean as already reported [24,70] but a limited ability to systemically infect plants of this host (confirmed by PCR amplification and sequencing) was observed for the recombinant TYLCSV described here (Table 3). This will merit further study. As expected from previous studies [24], the TYLCV isolate was able to efficiently systemically infect plants of tomato and common bean but not of *S. nigrum*. An isolate of the putatively parental TYLCSV strain Sar/Group 1 was not included in this comparative study because of the quarantine restrictions since isolates of this strain have not been reported in Spain (Table 1). Therefore, a closely similar biological behaviour is observed for isolate [ES-Mur-TY2-Tom-11] and the TYLCSV previously reported in Spain.

### 3.5. Behaviour of Isolate TYLCSV[ES-Mur-TY2-Tom-11] in TYLCD-Resistant Tomatoes

As differences in the ability to infect TYLCD-resistant tomatoes have been observed for some TYLCD-associated recombinant viruses [24,26], the effect of the resistance traits present in tomato cultivars with resistance genes *Ty-1*, *Ty-3*, *Ty-1/Ty-3*, *Ty-2* and *tcm-1* on the infection ability of the recombinant isolate characterised here was examined and compared to isolates of TYLCV (isolates of type and Mld strains) and TYLCSV (strain ES isolate) reported in Spain. The TYLCD resistance described in *Solanum habrochaites* EELM-889 [44] was also tested. As shown in Table 4, in the tomatoes susceptible to TYLCD (ty-Sks and ty-1S), 100% effective infection was observed with infected plants exhibiting typical TYLCD symptoms and AUSPC ranging from 60 to 100. In general, TYLCV induced more severe symptoms than TYLCSV in infected plants (higher AUSPC and DSI values). In the *Ty-1* heterozygous tomato line Ty-1F1, most of the plants became systemically infected with TYLCSV or TYLCV isolates but no symptoms were observed except for the isolate of the type strain of TYLCV that induced very mild symptoms (low AUSPC and DSI values). The *Ty-1*-heterozygous tomato line TY-Sk1 exhibited even better resistance than the *Ty-1*-homozygous TY-1R line, showing no infection except for TYLCV isolates which resulted in some infected plants. None of the virus-infected plants of the two latter tomato genotypes exhibited TYLCD symptoms of infection and faint hybridisation signals were observed for infected plants.

In the *Ty-3*-heterozygous tomato line TY-Sk3, all the virus isolates tested resulted into effective systemic infection, although TYLCD symptoms were observed only for the isolates of the Mld and type TYLCV strains, with 46 and 32% DSI, respectively. These results suggested that *Ty-3* gene might be overcome by TYLCV and only confers effective resistance to TYLCSV-like isolates. Interestingly, in contrast to the *Ty-1*-heterozygous tomato line TY-Sk1, in the tomato line TY-Sk13 that carry the *Ty-1* gene in heterozygous manner with the *Ty-3* gene, the *Ty-1*-derived resistance seemed to express less efficiently, as most of the plants exhibited systemic infection with TYLCSV or TYLCV and (mild) symptoms of infection (5.9% DSI) were observed for plants infected with the isolate of the TYLCV type strain.

Similar to the results of previous studies [33,71], the *Ty-2* homozygous resistant tomato line H24 exhibited effective resistance (no virus systemic accumulation and no symptoms) to the TYLCV type isolate, whereas the isolate of the Mld strain of TYLCV was fully infectious (100% incidence in inoculated plants) resulting into severe TYLCD symptoms (above 80% DSI). Comparable results were obtained for inoculations with isolates of the recombinant TYLCSV reported in this work and the TYLCSV (strain ES) used as control, which resulted to be highly infectious in this tomato genotype with severe symptoms (DSI values of 100%).

The TYLCD resistance present in *Solanum habrochaites* EELM-889 genotype shown to be controlled by one dominant and one recessive independent loci [44] behaved similarly to H24 against TYLCV isolates with complete resistance to the TYLCV type isolate whereas the TYLCV-Mld isolate resulted in highly effective infection with prominent TYLCD symptoms in infected plants (80% DSI). In contrast, EELM-889 plants showed a fairly effective control of both TYLCSV isolates, with only limited number of plants infected that exhibited mild symptoms of infection (DSI values of 53% and 46%).

Finally, plants with the recessive *tcm-1*-based resistance present in line TX-468 reported as effective to both bipartite begomoviruses and monopartite begomoviruses associated to TYLCD [46] exhibited intermediate resistance to either TYLCV or TYLCSV isolates with significant number of plants infected but with faint hybridisation signals and mild symptoms (low DSI values).

Therefore, in conclusion, the results obtained here suggested that no significant differences were observed in the behaviour of resistant tomato cultivars/lines and *Solanum habrochaites* EELM-889 against the recombinant isolate TYLCSV[ES-Mur-TY2-Tom-11] reported here when compared with an isolate of TYLCSV previously reported in Spain

## 4. Discussion

As for TYLCV and other begomoviruses [35,72,73], presence of taxonomically differentiated strains in the population of the TLCSV species is shown here. Although recognition of different strains was not done for TYLCSV in the recent begomovirus compilations of the *Geminiviridae* Study Group of the ICTV [68] or ICTV (current online ICTV Report on Virus Taxonomy, https://talk.ictvonline.org/ictv-reports/), the existence of different strains in the TYLCSV species was proposed in previous reports [69]. Here, the genetic diversity of TYLCSV was revisited and studied following current taxonomic criteria. A comprehensive analysis of full-length genome sequences available in databases for TYLCSV following the novel guidelines for begomovirus strain demarcation [68] indicated that isolates grouped into two major strains (named Sardinia, Sar and Spain, ES). Additionally, two phylogenetically differentiated groups are found present among isolates of the proposed TYLCSV-Sar strain that correspond to isolates that grouped in the Sicily (Sic) and Sardinia (Sar) strains previously suggested for TYLCSV [69]. Thus, with current criteria these latter isolates merge in only one taxonomically distinct strain for which the name Sar was conserved as it was the one associated with the oldest TYLCSV isolate available in databases [62,69]. This information is relevant as the presence of different strains in the TYLCSV population should be taken into account because it might have epidemiological or control consequences. In fact, pathological differences could exist between isolates of different begomovirus strains such as for TYLCV in which isolates of the strain Mild or of the type strain (also known as Israel, IL, in some reports) differ in their ability to overcome resistance traits in tomato genotypes [33,44]. Also, differences in the ecological adaptation of isolates of different strains might exist. This has been reported, for example, for isolates of the Mld strain of TYLCV that were rapidly displaced by isolates of the type TYLCV in Spanish epidemics suggesting a better ecological adaptation of the latter [52].

It is interesting to note that we have shown here that in addition to point mutation probably combined with selection and genetic drift, the evolution of the TYLCSV species seems to be driven by genetic exchanges achieved through inter-species recombination. Thus, isolates of strain Sar/Group 1 were shown to derive from a genetic exchange involving the IR and the 5′-end of the Rep gene, a region prone to recombination events in begomoviruses [74], for which a SACMV-related ancestor was detected. Maintenance of cognate 5′ C1-IR interactions (Rep-IR interactions) in this genetic exchange preserves biologically important intra-genome interactions [75]. Recombination is a frequent phenomenon in begomoviruses [22,26,76,77] and has profound effects in virus populations facilitating the rapid modular evolution by exchanges of genetic information that might be essential for virus adaptation to changing environmental conditions [22]. Recombination is expected to rapidly bring together favourable gene combinations to challenge, for example, host resistance [78]. Thus, among the multifunctional begomovirus proteins, several proteins have been shown to be involved into counteracting plant defences based on gene silencing [17]. Optimal combination of such proteins achieved through recombination could improve the adaptive capacity of a begomovirus to specific conditions. This has been suggested, for example, to be the basis of the ability of TYLCD-associated viruses to adapt to the *Ty-1* resistance [24,40]. Similarly, genome differences between the Mld and the type strains of TYLCV acquired by recombination [79] have also been suggested to underlie their differential ability to overcome resistance traits [44].

We have characterised here a TYLCSV isolate collected from Spanish epidemics for which a mosaic composition of the genome putatively driven by a TYLCSV inter-strain recombination is suggested. The phylogenetic relationship incongruences detected depending on the genomic region compared supported a modular evolution [21], with two genomic regions with distinct evolutionary histories. Incongruence in the topology of phylogenetic trees derived from different genomic regions is generally considered as evidence of recombination [80]. The recombination nature of this isolate was statistically supported, concluding that it is a recombinant putatively resulted from a genetic exchange between isolates of the two different strains (strain Sar/Group 1 and strain ES) of TYLCSV. As far as we know, this would be the first report of an isolate derived from an inter-strain recombination in TYLCD-associated viruses for which inter-species recombination involving ancestors belonging to different begomovirus species were reported [24,26,38,70,79]. Nevertheless, as the minor region involved in the genetic exchange observed in this novel recombinant isolate coincides phylogenetically with the region of SACMV origin of strain Sar/Group 1 isolates (see above and cf. Figure 3 and Figure 4), we cannot rule out that this fragment was acquired directly by a strain ES isolate from an ancestor phylogenetically related to the SACMV that donated this region to strain Sar/Group 1 isolates. In either case, the detection of a novel recombinant in Spanish epidemics is an important observation that further supports that as shown for other related begomoviruses, recombination plays a central role in the evolution of TYLCD-associated viruses [22] and the dynamism of their populations. The latter is a challenge for the control of their impact in tomato production. Also, as no geographical overlapping has been described for isolates of strains ES and strain Sar of TYLCSV (confer Table 1), the existence of a recombinant putatively combining sequences from these two viruses implies that virus isolates sharing genome sequences of their ancestors might have co-existed in nature in a mixed infection, which is a prerequisite for recombination to occur. Therefore, additional samplings and characterisation studies are needed to better understand the geographical distribution of isolates of TYLCSV strains and the complexity of the TYLCSV population in the Mediterranean Basin.

Recombination has been reported that can have relevant phenotypic consequences, such as increased virulence or changes in host range [81,82]. No significant host range differences were observed for the recombinant isolate reported here respect to TYLCSV isolates of the strain ES previously reported in Spain. Nevertheless, the possible ability to infect common bean not observed for the latter isolates [24,70] will merit further study as it can have unknown epidemiological consequences. In this sense, it should be highlighted that increased pathogenicity in common bean was suggested to be involved in the increased ecological fitness of TYLCV versus TYLCSV in Spanish epidemics [51]. No significant differences were observed, however, in the ability to infect resistant tomato genotypes for the recombinant TYLCSV isolate characterised here with respect to the TYLCSV already present in Spain. This might suggest the stability of the genes used commercially in the TYLCD-resistant tomato cultivars if this recombinant variant spreads in the TYLCD-associated population. However, it should be taken in mind that small alterations in the begomovirus sequence derived from recombination events can determine increased ecological adaptation in resistant cultivars as recently observed for TYLCV in Morocco [26]. Interestingly, the results of challenging resistant tomatoes with different TYLCSV and TYLCV isolates demonstrates the existence of a variable virus x plant genotype interaction. This has already been observed for TYLCD-associated viruses [33,44,71,83,84,85] and was suggested to be involved in the evolution of the TYLCD-associated virus population [86]. It is noteworthy that the results obtained here supported the different ability of alleles *Ty-3* and *Ty-1* of the same resistance locus to control TYLCD-associated viruses as already suggested [43,87]. Moreover, differences were also observed for different *Ty-1*-based resistances that might suggest additional allele differences. Nevertheless, as isogenic lines were not available, background effect differences cannot be discarded as resistance phenotype might depend on the genetic background [88]. The lower resistance effectiveness of the *Ty-1/Ty-3* line TY-Sk13 respect to the *Ty-1* heterozygous line TY-Sk1 might suggest negative interaction of *Ty-1* and *Ty-3* genes which might merit further study. But, again, the effect of background differences cannot be discarded.

We conclude, therefore, that genetically complex relationships are observed in the TYLCSV population in which recombination events are driving evolution. Novel TYLCSV recombinants can appear such as the one characterised here, with new biological properties of unknown phytopathological consequences that can complicate TYLCSV control. All this reinforces the importance of monitoring virus populations present in the TYLCD epidemics to detect relevant alterations that could challenge control strategies.

## Figures and Tables

**Table 1:** Tomato yellow leaf curl Sardinia virus (TYLCSV) isolates for which the full-length genome sequence was available in databases as of the 19th of September 2018, including information about the GenBank accession number of the nucleotide sequence of the genome, the host species from which they were derived, the geographical origin and collection date; the isolate characterised in this study is highlighted in bold letters.

Tomato Yellow Leaf Curl Sardinia Virus Isolate	Acronym	GenBank Accession Number	Host Species	Geographical Origin	Collection Date	Reference
Tomato yellow leaf curl Sardinia virus-[Spain-Murcia 1-1992]	TYLCSV-[ES-Mur1-92]	Z25751	*Solanum lycopersicum*	Spain	1992	[50]
Tomato yellow leaf curl Sardinia virus-[Spain-Almeria 2-1992]	TYLCSV-[ES-Alm2-92]	L27708	*Solanum lycopersicum*	Spain	1992	[61]
Tomato yellow leaf curl Sardinia virus-[Spain-Canary]	TYLCSV-[ES-Can]	AJ519675	*Solanum lycopersicum*	Spain	NA	NA ^a^
Tomato yellow leaf curl Sardinia virus-[Spain-Malaga-TY73-Pepper-2003]	TYLCSV-[ES-Mlg-TY73-Pep-03]	KC953603	*Capsicum annuum*	Spain	2003	NA
**Tomato yellow leaf curl Sardinia virus-[Spain-Murcia-TY2-Tomato-2011]**	**TYLCSV-[ES-Mur-TY2-Tom-11]**	**KC953604**	***Solanum lycopersicum***	**Spain**	**2011**	**Present study**
Tomato yellow leaf curl Sardinia virus-[Italy-Sardinia-1988]	TYLCSV-[IT-Sar-88]	X61153	*Solanum lycopersicum*	Italy	1988	[62]
Tomato yellow leaf curl Sardinia virus-[Italy-Sicily-1991]	TYLCSV-[IT-Sic-91]	Z28390	*Solanum lycopersicum*	Italy	1991	[63]
Tomato yellow leaf curl Sardinia virus-[Italy-Sicily-2004]	TYLCSV-[IT-Sic-04]	GU951759	*Solanum lycopersicum*	Italy	2004	[64]
Tomato yellow leaf curl Sardinia virus-[Portugal-Algarve 6-2001]	TYLCSV-[PT-Alg6-01]	JN859136	*Solanum lycopersicum*	Portugal	2001	NA
Tomato yellow leaf curl Sardinia virus-[Portugal-Algarve 2-2001]	TYLCSV-[PT-Alg2-01]	JN859134	*Solanum lycopersicum*	Portugal	2001	NA
Tomato yellow leaf curl Sardinia virus-[Morocco-22-2001]	TYLCSV-[MA-22-01]	LN846595	*Solanum lycopersicum*	Morocco	2001	[40]
Tomato yellow leaf curl Sardinia virus-[Morocco-Agadir-2002]	TYLCSV-[MA-Aga-02]	AY702650	*Solanum lycopersicum*	Morocco	2002	[65]
Tomato yellow leaf curl Sardinia virus-[Morocco-8-1-2005]	TYLCSV-[MA-8-1-05]	LN846596	*Solanum lycopersicum*	Morocco	2005	[40]
Tomato yellow leaf curl Sardinia virus-[Morocco-S15-2010]	TYLCSV-[MA-S15-10]	LN846597	*Solanum nigrum*	Morocco	2010	[40]
Tomato yellow leaf curl Sardinia virus-[Morocco-5a-2012]	TYLCSV-[MA-5a-12]	LN846598	*Solanum lycopersicum*	Morocco	2012	[40]
Tomato yellow leaf curl Sardinia virus-[Tunisia-Bkalta 3-2002]	TYLCSV-[TN-Bka3-02]	AY736854	*Solanum lycopersicum*	Tunisia	2002	[66]
Tomato yellow leaf curl Sardinia virus-[Jordan-2011]	TYLCSV-[JO-11]	JX131285	*Sinapis arvensis*	Jordan	2011	[67]
Tomato yellow leaf curl Sardinia virus-[Israel-Rehovot-2005]	TYLCSV-[IL-Reh-05]	DQ845787	NA	Israel	2005	NA

^a^ NA, data not available.

**Table 2:** Recombination event detected in the genome of tomato yellow leaf curl Sardinia virus (TYLCSV) isolate [ES-Mur-TY2-Tom-11] and isolates of closely-related begomoviruses putatively involved in the recombination (the GenBank accession numbers of the nucleotide sequences are shown between brackets).

Recombinant Sequence	Major Parent	Minor Parent	Breakpoints ^a^		Methods ^b^	*p*-Value ^c^
			Begin	End		
TYLCSV-[ES-Mur-TY2-Tom-11] (KC953604)	TYLCSV-[ES-Mur1-92] (Z25751)	TYLCSV-[IT-Sar-88] (X61153)	1951	56	RGBMCT	1221 × 10^−2^

^a^ Positions in the recombinant sequence; ^b^ only events detected with three or more methods of RDP4 program [60] with acceptable p-Value were considered credible evidence of recombination. R = RDP, G = GENECONV, B = BootScan, M = MaxChi, C = Chimaera, T = 3Seq; ^c^ the reported *p*-value is the highest obtained for that region.

**Table 3:** Infectivity in plants of *Solanum lycopersicum* (tomato cv. Moneymaker), *Solanum nigrum* and *Phaseolus vulgaris* (common bean) of the tomato yellow leaf curl Sardinia virus (TYLCSV) isolate characterised in this study (TYLCSV-[ES-Mur-TY2-Tom-11]) and Spanish control isolates of the strain Spain (ES) of TYLCSV (isolate TYLCSV-[ES-Mur1-92]) and of the strain Mild (Mld) of tomato yellow leaf curl virus (TYLCV) (isolate TYLCV-Mld-[ES-72-97]) ^a^.

Plant Species	TYLCSV-[ES-Mur-TY2-Tom-11]		TYLCSV-[ES-Mur1-92]		TYLCV-Mld[ES-72-97]	
	Assay 1	Assay 2	Assay 1	Assay 2	Assay 1	Assay 2
*Solanum nigrum*	10/10 ^b^	8/9	10/10	9/9	0/8	0/9
Common bean	0/10	1/9	0/10	0/9	7/8	9/9
Tomato (Moneymaker)	10/10	10/10	10/10	9/10	9/10	10/10

^a^ The plants were inoculated with infectious clones by *Agrobacterium tumefaciens*-mediated inoculation. For each virus/plant host combination, about 10 seedlings were inoculated per assay at 3–4 leaf growth stage. Inoculated plants were analysed for the presence of virus in newly-emerged young leaves by squash blot hybridisation at 30 days post inoculation; ^b^ number of plants systemically infected/number of plants inoculated.

**Table 4:** Infectivity in plants of different cultivars and genotypes of tomato and of *Solanum habrochaites* EELM-889 of the tomato yellow leaf curl Sardinia virus (TYLCSV) recombinant isolate characterised in this study (TYLCSV-[ES-Mur-TY2-Tom-11]) and of control isolates from Spain of the strain Spain (ES) of TYLCSV (TYLCSV-[ES-Mur1-92]) and of the type and Mild (Mld) strains of tomato yellow leaf curl virus (TYLCV) (isolates TYLCV-[ES-Alm-Pep-99] and TYLCV-Mld-[ES-72-97], respectively); the area under the symptom progress curve (AUSPC) and the disease severity index (DSI) at 42 days post inoculation (dpi) are indicated.^a.^

	TYLCSV-[ES-Mur-TY2-Tom-11]		TYLCSV-[ES-Mur1-92]		TYLCV-Mld[ES-72-97]		TYLCV-[ES-Alm-Pep-99]	
	No of Plants Infected/No Inoculated	AUSPC (DSI)	No of Plants Infected/No Inoculated	AUSPC (DSI)	No of Plants Infected/No Inoculated	AUSPC (DSI)	No of Plants Infected/No Inoculated	AUSPC (DSI)
ty-SkS	10/10	70.4 ± 4.1 (98%)	10/10	59.2 ± 0.8 (90%)	10/10	100.1 ± 0.7 (100%)	8/10	100.6 ± 0.9 (100%)
TY-Sk1 (*Ty-1*/*ty-1*)	0/9	0 (0%)	0/10	0 (0%)	3/10 *	0 (0%)	5/10 *	0 (0%)
TY-Sk3 (*Ty-3*/*ty-3*)	7/10	0 (0%)	9/10	0 (0%)	10/10	40.7 ± 4.7 (46%)	6/10	43.4 ± 8.7 (32%)
TY-Sk13 (*Ty-1*/*Ty-3*)	8/10	0 (0%)	8/10	0 (0%)	9/10	0 (0%)	7/10	11.8 ± 0.6 (5.9%)
ty-1S (*ty-1*/*ty-1*)	10/10	64.4 ± 2.6 (100%)	10/10	43.1 ± 5.1 (78%)	10/10	84.0 ± 0.9 (100%)	6/10	72.9 ± 0.8 (90%)
Ty-1F1 (*Ty-1/ty-1*)	9/10	0 (0%)	7/10	0 (0%)	9/10	0 (0%)	8/10	19.3 ± 8.3 (15%)
Ty-1R (*Ty-1*/*Ty-1*)	2/10 *	0 (0%)	2/10 *	0 (0%)	10/10	0 (0%)	4/10 *	0 (0%)
*S. habrochaites* EELM-889	4/10	17.9 ± 3.1 (53%)	5/10	38.2 ± 14.3 (46%)	9/10	64.0 ± 0.8 (70%)	0/10	0 (0%)
H24 (*Ty-2*/*Ty-2*)	10/10	85.4 ± 2.3 (100%)	10/10	82.6 ± 0.6 (100%)	10/10	69.0 ± 1.2 (80%)	0/10	0 (0%)
TX 468-RG	9/10 *	9.5 ± 3.2 (12%)	10/10 *	19.9 ± 3.9 (20%)	8/10 *	15.7 ± 5.1 (15%)	5/10 *	10.2 ± 3.4 (14%)

^a^ For each virus/genotype combination, about 10 seedlings were inoculated with infectious clones at 4–5 leaf growth stage by *Agrobacterium tumefaciens*-mediated inoculation. Inoculated plants were analysed at 15, 21, 28, 35 and 42 dpi for the presence of virus in newly-emerged young leaves by squash blot hybridisation. The AUSPC was calculated from symptom data collected for infected plants at 15, 21, 28, 35 and 42 dpi.^.^ Asterisk (*) highlight infections that resulted in faint hybridisation signals.

## Supplementary Materials

The following are available online at http://www.mdpi.com/1999-4915/11/1/45/s1, Table S1: Recombination event detected in tomato yellow leaf curl Sardinia virus (TYLCSV) strain Sar/Group 1 isolates.
