# Supplementary material for: Tomato Yellow Leaf Curl Sardinia Virus, a Begomovirus Species Evolving by Mutation and Recombination: A Challenge for Virus Control
Source: Viruses. 2019 Jan 9;11(1):45. doi: 10.3390/v11010045 (PMC6356960; doi:10.3390/v11010045)
Supplement: Supplementary file 1 [file viruses-11-00045-s001.pdf]

Supplementary Material Table S1. Recombination event detected in tomato yellow leaf curl Sardinia virus (TYLCSV) strain Sar/Group 1 isolates exemplified for isolate [IT-Sar-88] involving a major parent from strain Sar/Group 2 (exemplified with isolate [IT-Sic-91] and a minor parent from the begomovirus South African cassava mosaic virus (SACMV) (isolate [MG-MG718A2-11]) (the Gen Bank accession numbers of the nucleotide sequences are shown between brackets).

Recombinant Sequence	Major Parent	Minor Parent	Breakpoints[a]		Methods[b]	p-Value[c]
			Begin	End		
TYLCSV-[IT-Sar-88] (X61153)	TYLCSV-[IT-Sic-91] (Z28390)	SACMV-[MG-MG718A2-11] (KL888094)	2416	2744	RGBMCT	$4{,}777 \times 10^{-11}$

[a] Positions in the recombinant sequence; [b] methods of RDP4 program (Martin et al., 2015) with acceptable p-Value. R = RDP, G = GENECONV, B = BootScan, M = MaxChi, C = Chimaera, T = 3Seq; [c] the reported p-value is the highest obtained for that region
